# Performance of SARS-CoV-2 Antigen-Detecting Rapid Diagnostic Tests for Omicron and Other Variants of Concern

**DOI:** 10.3389/fmicb.2022.810576

**Published:** 2022-05-10

**Authors:** Dàlia Raïch-Regué, Jordana Muñoz-Basagoiti, Daniel Perez-Zsolt, Marc Noguera-Julian, Edwards Pradenas, Eva Riveira-Muñoz, Neus Giménez, Assumpta Carabaza, Francesc Giménez, Verónica Saludes, Elisa Martró, Neus Robert, Ignacio Blanco, Roger Paredes, Lidia Ruiz, Ester Ballana, Bonaventura Clotet, Julià Blanco, Nuria Izquierdo-Useros

**Affiliations:** ^1^IrsiCaixa AIDS Research Institute, Germans Trias i Pujol Research Institute (IGTP), Badalona, Spain; ^2^AIDS and Infectious Deseases Department, University of Vic–Central University of Catalonia (UVic-UCC), Vic, Spain; ^3^Infectious Disease Networking Biomedical Research Center, Centro de Investigación Biomédica en Red de Enfermedades Infecciosas (CIBERINFEC), Carlos III Health Institute, Madrid, Spain; ^4^Nesapor Europa, Premià de Dalt, Spain; ^5^Laboratori Clínic Metropolitana Nord, Department of Microbiology, Germans Trias i Pujol University Hospital and Research Institute (IGTP), Badalona, Spain; ^6^Epidemiology and Public Health Networking Biomedical Research Centre (CIBERESP), Instituto de Salud Carlos III, Madrid, Spain; ^7^Department of Emergency, Germans Trias i Pujol University Hospital and Research Institute (IGTP), Badalona, Spain

**Keywords:** SARS-CoV-2, diagnosis, antigen-detecting rapid diagnostic tests, variants of concern, nucleocapsid (N)

## Abstract

The SARS-CoV-2 antigen-detecting rapid diagnostic test (Ag-RDTs) is an easy-to-use diagnostic tool to identify the contagious individuals and reduce the new infections. However, to be effective, Ag-RDTs require the detection of distinct variants of concern (VOC) with high analytical sensitivity. Here, we found that the VOC diverge at the nucleocapsid protein used by four commercial Ag-RDTs for the viral detection. Relative to the original D614G variant, there was a 10-fold loss of detection for the Delta and Alpha variants in certain Ag-RDTs, a reduction above the threshold required to isolate the viable virus. However, Beta and Omicron variants did not lose the detection capacity. As the new VOC arise, successful contact tracing requires continuous monitoring of Ag-RDTs performance.

## Introduction

The use of antigen-detecting rapid diagnostic tests (Ag-RDTs) is a valuable strategy for the diagnosis and control of infectious diseases such as Malaria or HIV-1/AIDS. The recent WHO guidelines recommend Ag-RDTs for infant HIV diagnosis and viral load monitoring in resource limited settings (World Health Organization, 2021). This approach could also represent a cost-effective measure to monitor the SARS-CoV-2 infection if the implementation adheres to the guidelines proposed by the WHO (World Health Organization, 2020). The recent evidence from the observational and randomized controlled open-label trials support the value of SARS-CoV-2 Ag-RDTs for monitoring the infections at mass gathering events (Llibre et al., 2021; Revollo et al., 2021). The prior performance studies also suggested their usefulness for the screening of asymptomatic individuals, particularly in communities with high prevalence (Alemany et al., 2021).

Currently, Ag-RDTs are used not only by the healthcare providers but also by the general public for consumer use. The kinetics of SARS-CoV-2 replication in the upper respiratory tract is not fully characterized and may vary depending on the variants of concern (VOC) (Li et al., 2021). Yet, the period when the individuals are presumed to be more contagious is thought to last for over 10 days (Wölfel et al., 2020). Identifying the early exponential viral growth phase when individuals achieve higher viral loads, even if they are asymptomatic, and lack neutralizing immune responses, is critical to avoid the novel infections. Indeed, the viral loads above 10^6^ viral RNA copies/ml in index cases are associated with a higher transmission rates among their contacts (Marks et al., 2021). Evidence also suggests that the identification of asymptomatic or early infection cases is critical in breakthrough infections in the vaccinated individuals, which also achieve a high viral loads (Lange et al., 2021; Riemersma et al., 2021). Of note, the Delta variant has been successfully isolated *in vitro* from nasal swabs of vaccinated individuals with more than 10^6^ RNA copies/ml (Riemersma et al., 2021), the threshold previously reported for the isolation of replication-competent viruses for the other variants (Wölfel et al., 2020). The Delta variant is more contagious, as over 80% of the samples analyzed in oropharyngeal swabs at the moment of diagnosis had viral loads above that threshold, in contrast to the 20% observed for other variants (Li et al., 2021).

The expansion of Ag-RDTs to diverse geographical areas implies that these tests are being used in communities where distinct VOC coexist. The Ag-RDTs were originally designed for the detection of the Wuhan SARS-CoV-2 initial strain. This raises the possibility that the antigen recognition capacity of these Ag-RDTs may now be compromised due to the surge of mutations in distinct VOC. The purpose of this study was to compare the analytical detection capacity of different Ag-RDTs for distinct SARS-CoV-2 VOC grown in the laboratory, which were used as standards to examine test performance. We report a 10-fold reduction in the detection capacity for the Alpha variant in the four Ag-RDTs tested in our study. For the Delta variant, a similar reduction was found in two of the commercial tests analyzed herein, while for the Zeta variant, this was observed in one Ag-RDTs. The detection capability was not lost for the Beta and Omicron variants across the 10-fold dilution range we tested.

## Materials and Methods

### Biosafety Approval

The biologic biosafety committee of the Germans Trias i Pujol Research Institute (IGTP) approved the execution of SARS-CoV-2 experiments at the BSL3 laboratory of the Center for Bioimaging and Comparative Medicine (CSB-20-015-M3).

### Virus Isolation and Sequencing

The SARS-CoV-2 VOC were isolated from clinical nasopharyngeal swabs, as described in a previous study (Rodon et al., 2021), and subsequently grown in Vero E6 cells. The following SARS-CoV-2 variants with deposited genomic sequence at the Global Initiative on Sharing Avian Influenza Data (GISAID) repository^1^ were tested: B.1 (D614G) isolated in Spain in March 2020 (EPI_ISL_510689); four VOC isolated in Spain from January to February 2021: Alpha or B.1.1.7 (EPI_ISL_1663569), Beta or B.1.351 (originally detected in South Africa; EPI_ISL_1663571), Zeta or P.2 (originally detected in Brazil; EPI_ISL_1831696), Delta or B.1.617.2 (originally detected in India; EPI_ISL_3342900), and Omicron or B.1.1.529 isolated in Spain in December 2021 (originally detected in South Africa; EPI_ISL_8151031). The genomic sequencing was performed from the viral supernatant by using the standard ARTIC v3- or v4-based protocols followed by Illumina sequencing (Pillay et al., 2022). A raw data analysis was performed by viralrecon pipeline^2^ while consensus sequence was called using samtools/ivar at the 75% frequency threshold.

### Nucleocapsid Detection by Enzyme-Linked ImmunoSorbent Assay

The amount of SARS-CoV-2 nucleoprotein in viral stocks was measured with SARS-CoV-2 nucleocapsid protein high-sensitivity quantitative enzyme-linked immunosorbent assay (ELISA) (ImmunoDiagnostics) according to the manufacturer’s protocol. This commercial kit is based on a mixture of polyclonal antibodies that is able to cross-react and detect the nucleocapsid of SARS-CoV as well. Thus, this system is suitable for quantifying different SARS-CoV-2 variants.

### Quantitative Polymerase Chain Reaction

The RNA extraction was performed by using Viral RNA/Pathogen Nucleic Acid Isolation Kit (Thermo Fisher Scientific), optimized for a KingFisher instrument (Thermo Fisher Scientific), following manufacturer’s instructions. The reverse transcription and polymerase chain reaction (PCR) amplification was based on the 2019 Novel Coronavirus (2019-nCoV) Real-Time Reverse Transcription Polymerase Chain Reaction (RT-PCR) Diagnostic Panel Guidelines and Protocol developed by the American Center for Disease Control and Prevention (CDC, 2019). Briefly, a 20 μl PCR reaction was set up, which was containing 5 μl of RNA, 1.5 μl of primers and probe targeting the N2 region (2019-nCov CDC EUA Kit, Integrated DNA Technologies), and 10 μl of GoTaq 1-Step RT-qPCR (Promega). The thermal cycling was performed at 50°C for 15 min for the reverse transcription, followed by 95°C for 2 min and then 45 cycles of 95°C for 10 s, 56°C for 15 s and 72°C for 30 s in the Applied Biosystems 7,500 or QuantStudio5 R-T PCR instruments (Thermo Fisher Scientific). The N_2_ primers have the following positions in the reference nucleocapsid sequence: N2 Forward: 29140-29164 and N2 Reverse: 29229-29248. These two positions are not affected by the specific mutations identified in the SARS-CoV-2 variants used in this study. For absolute quantification, a standard curve was built using 1/5 serial dilutions of a SARS-CoV2 plasmid (2019-nCoV_N_Positive Control, 200 copies/μl, Integrated DNA Technologies) and run in parallel in all PCR determinations. The viral load of each sample was determined in triplicate and mean viral load (in copies/ml) was extrapolated from the standard curve and corrected by the corresponding dilution factor.

### The Ag-RDT for SARS-CoV-2

The following available commercial tests were compared: Nesapor (Mareskit ^®^) and Roche (SD biosensor) for nasal swabs, and Siemens Healthineers (Clinitest ^®^) and Abbott (Panbio™ COVID-19 Ag Rapid Test) for nasopharyngeal and nasal swabs. These brands were selected as the most representative ones used in the geographical area of our hospital. We used serial dilutions of direct viral culture supernatant, as previously reported in the standardized protocol for the evaluation of limit of detection in Ag-RDTs specific for SARS-CoV-2 antigens by the Department of Health and Social Care in United Kingdom^3^. The viral stocks were serially diluted 1/10 in phosphate buffer saline (PBS), and tested with the indicated SARS-CoV-2 antigen kits by mixing 10 μl of the viral dilution with 190 μl of the corresponding test lysis buffer (1:19 ratio), or by mixing 100 μl of the viral dilution with 100 μl of lysis buffer (1:1 ratio). For each detection, four drops of the indicated mixtures were added to each antigen test and incubated for 15 min before the visual readout. Duplicates were made for all Ag-RDTs, VOC, and dilutions. The highest dilution detected as positive by each Ag-RDTs was recorded as the lower detection capacity for each viral variant. The mean weight of mucus sample collected per hyssop for the Mareskit test is 21 mg. We therefore calculated that a mean of 21 μl of this waterish sample would be diluted in 400 μl of lysis buffer per test, what yields a 1:19 dilution, which was considered the sample to buffer ratio more similar to physiological settings.

## Results

**FIGURE 1:**
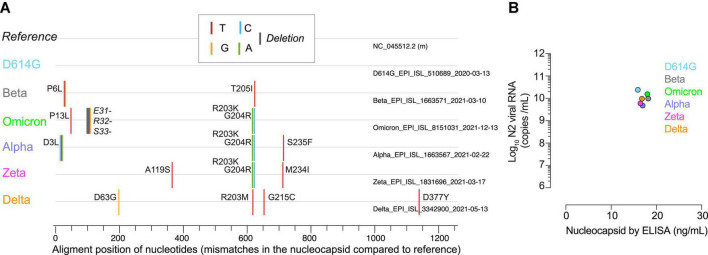
Nucleocapsid mismatches and quantification of the SARS-CoV-2 variants. **(A)** Nucleotide mismatches or deletions (represented by the colored lines of the legend) and amino acid changes indicated by codon position sequenced in the nucleocapsid of distinct SARS-CoV-2 variants as compared to the original Wuhan virus (as reference). **(B)** Nucleocapsid concentration measured by qPCR (log_10_ of viral N2 RNA copies/ml) and ELISA (ng of nucleocapsid/ml) for all variants analyzed at the first dilution tested in the Ag-RDTs.

Many Ag-RDTs rely on viral nucleocapsid recognition to detect SARS-CoV-2 infection as it is the most abundant viral protein. The emergence of new VOC with particular variations in the nucleocapsid could impact the lower detection limit of these tests. To explore this possibility, we first analyzed whether distinct SARS-CoV-2 variants isolated and grown in cellular culture in the laboratory displayed variations in the nucleocapsid gene sequence as compared to the reference sequence of Wuhan-Hu-1 (Figure 1A). While the D614G variant showed no changes, the Beta VOC displayed 2 non-synonymous mutations, the Omicron displayed 3 non-synonymous mutations plus one deletion, and the Alpha, Zeta, and Delta variants contained up to 4 non-synonymous changes (Figure 1A). As we detected non-synonymous variations in the nucleocapsid of these variants, we next assessed if they could impact viral recognition *via* Ag-RDTs. To explore this possibility, we compared the performance and analytical detection capacity of different commercial Ag-RDTs to detect each of the VOC mentioned here. The measurements assessed with both qPCR of the N2 viral RNA and ELISA of the viral nucleocapsid protein allowed us to use similar concentrations of each of the distinct SARS-CoV-2 variants in the following experiments (Figure 1B).

**FIGURE 2:**
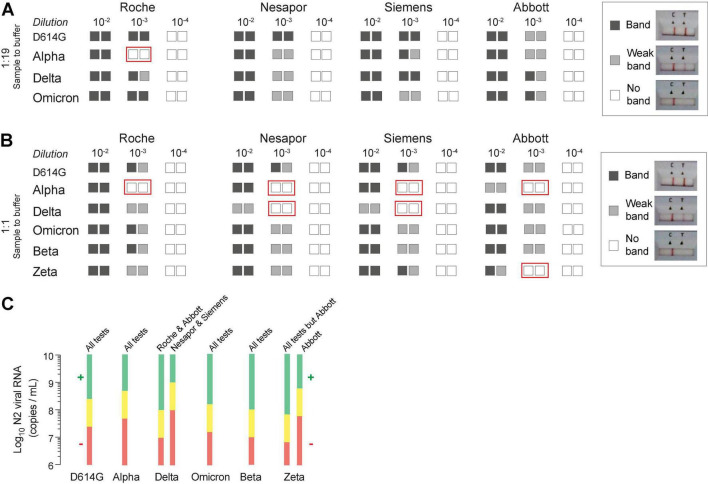
Comparative detection of SARS-CoV-2 variants by four commercial Ag-RDTs and correlation with nucleocapsid copies per ml. **(A)** Detection of 4 VOC using a 1:19 ratio of sample-to-buffer volume (10 μl:190 μl). **(B)** Detection of six variants using a 1:1 ratio of sample-to-buffer volume (100 μl:100 μl). The results for 1/10 viral dilutions tested in duplicate are indicated for each variant and commercial Ag-RDTs. Red boxes indicate variant-Ag-RDTs combination with reduced detection capacity as compared to D614G. **(C)** The detection range shown as values of the viral N2 RNA (in log_10_ of copies/ml). The range above the first 10-fold dilution with positive results of detection is shown in green, while the range below the first 10-fold dilution with negative results is depicted in red for each Ag-RDTs and every variant at the ratio 1:1.

The viral stocks with comparable nucleocapsid concentration measured with both techniques were serially diluted and used to test four commercial Ag-RDTs in parallel, to identify the last 10-fold dilution at which the tests yielded positive results (Figure 2). Assuming a collection volume of 10 μl for nasal and nasopharyngeal samples, the comparison was performed mixing 10 μl of variants with 190 μl of the respective buffer from each test (1:19 ratio; Figure 2A). This situation most likely resembles the sample dilution achieved with clinical samples. As compared to D614G, all variants were equally detected, but there was a 10-fold reduced detection for the Alpha variant in one commercial test (Figure 2A, red boxes). However, when we assumed a collection volume of 100 μl for nasal and nasopharyngeal samples, the detection capacity was more affected (Figure 2B). When we mixed 100 μl of each variant with 100 μl of the corresponding buffer (1:1 ratio), commercial Ag-RDTs had different detection capacities depending on the variant analyzed, and none of them performed equally for all of the variants (Figure 2B). All four commercial tests had a 10-fold reduced detection of the Alpha variant compared to D614G (Figure 2B, red boxes). The detection of the Delta variant was reduced 10-fold in Nesapor and Siemens Ag-RDTs, while the Zeta variant detection was decreased in Abbott test (Figure 2B, red boxes). The Beta and Omicron variants did not reduce the detection capability in any of the Ag-RDTs tested at the 10-fold dilutions assayed. We next represented the 10-fold detection capacity range of the different variants by distinct Ag-RDTs at a 1:1 sample to buffer ratio using the concentration of N2 viral RNA measured by qPCR (Figure 2C). All of the values found were above 10^6^ RNA copies/ml, which is the threshold required to isolate replication-competent virus from nasopharyngeal swabs in distinct variants (Wölfel et al., 2020; Riemersma et al., 2021).

Overall, these results highlight that commercial Ag-RDTs displayed different detection capacity for distinct VOC, which were specific for each of the tests. These findings are in line with the differences detected in the nucleocapsid sequence (Figure 1A), as variants such as D614G, without non-synonymous changes, or Beta or Omicron with up to three non-synonymous changes were equally detected by all Ag-RDTs tested herein. However, at least three Ag-RDTs showed a decreased detection capacity for the Zeta, Delta and Alpha variants (Figure 2B), which accumulated four non-synonymous changes (Figure 1A). Thus, some of the amino acid substitutions shown in Figure 1A will most likely affect the affinity of the antibodies used by distinct Ag-RDTs, decreasing their capacity to detect the nucleocapsid antigen.

## Discussion

The rapid, inexpensive, accessible, and easy-to-use approach of Ag-RDTs is arising as a critical tool for the early identification of the most infectious COVID-19 cases. The controlled clinical trials have already shown that this strategy can help to organize safe mass gathering events with additional protective measures, such as mask usage and adequate ventilation (Llibre et al., 2021; Revollo et al., 2021). Yet, the utility of these tests relies on their capacity to detect infected individuals throughout their period of contagiousness, which varies along the natural course of infection (Wölfel et al., 2020). This detection may be, however, hindered if the detection of circulating VOC is impaired, as we found here in all of the commercial Ag-RDTs analyzed for at least one of the variants tested.

The appearance of the new VOC with nucleotide changes throughout the viral genome may result in amino acid substitutions and conformational changes that may affect the viral nucleocapsid protein. These variations may jeopardize the testing capacity if analytical sensitivity is compromised. That is the reason why we examined the performance of different commercial Ag-RDTs in front of distinct VOC, which were used as standards for comparative detection. In this evaluation, we found a 10-fold reduction in the detection capacity for the Delta variant in two commercial Ag-RDTs, and for the Alpha variant in all the commercial Ag-RDTs analyzed at the most extreme conditions tested (ratio 1:1). These results suggest that the specific detection antibodies used for nucleocapsid recognition by each Ag-RDTs may display different affinities depending on the variant tested. The mutations affecting the recognition capacity of the nucleocapsid by Ag-RDTs depend on the nature of the capture antibody used by each test. Thus, the viral mutations cannot entirely predict the universal performance of Ag-RDTs. To provide a predictive efficacy, it would be important to know the viral epitopes recognized by the capture antibodies used by each commercial test. Here, we found that mutations of certain variants affected the detection capacity by up to 10-fold. Of note, this reduction is above the threshold required to isolate replication-competent virus from nasopharyngeal swabs (Riemersma et al., 2021). Hence, the reduction of the SARS-CoV-2 detection by some Ag-RDTs could hamper the identification of contagious individuals.

Early notified contacts could use commercial Ag-RDTs to monitor infection and contagiousness status over time. The isolation for positive individuals is needed until they produce high titers of neutralizing antibodies, viral load declines, infectiousness is reduced, and Ag-RDTs no longer detect the virus. Yet, for this strategy to succeed, Ag-RDTs will need to detect the virus as early as possible upon infection. This is particularly relevant as viral shedding may precede symptoms onset (Ge et al., 2021) and even peak earlier than previously reported for some particular VOC such as the Delta variant (Li et al., 2021). The 10-fold reduction in viral detection capacity of particular variants by commercial Ag-RDTs identified herein warrants future test performance surveillance. The VOC circulating in a geographical area at a given time may favor the usage of certain Ag-RDTs with the highest detection capacity for the early identification of contagious cases by that VOC.

While all Ag-RDTs tested herein are useful for current VOC detection, effective clinical and public implementation of these rapid tests will require a careful and constant monitoring of analytical sensitivity as new VOC arise.

## Data Availability Statement

The datasets presented in this study can be found in online repositories. The names of the repository/repositories and accession number(s) can be found below: http://gisaid.org, EPI_ISL_510689, http://gisaid.org, EPI_ISL_1663569, http://gisaid.org, EPI_ISL_1663571, http://gisaid.org, EPI_ISL_1831696, and http://gisaid.org, EPI_ISL_3342900.

## Author Contributions

DR-R, JM-B, NG, AC, FG, BC, JB, and NI-U conceived and designed the experiments. DR-R, JM-B, DP-Z, MN-J, EP, ER-M, VS, EM, and NR performed experiments. DR-R, JM-B, DP-Z, MN-J, EP, ER-M, NG, AC, FG, VS, EM, NR, IB, RP, LR, EB, BC, JB, and NI-U analyzed and interpreted the data. DR-R, JM-B, JB, and NI-U wrote the manuscript. All authors contributed to the article and approved the submitted version.

## Conflict of Interest

NG, AC, and FG were employed by company Nesapor Europa. The remaining authors declare that the research was conducted in the absence of any commercial or financial relationships that could be construed as a potential conflict of interest. The authors declare that this study received funding from Nesapor. The funder had the following involvement in the study: sponsored the research activity and contributed with the input of their employees.

## Publisher’s Note

All claims expressed in this article are solely those of the authors and do not necessarily represent those of their affiliated organizations, or those of the publisher, the editors and the reviewers. Any product that may be evaluated in this article, or claim that may be made by its manufacturer, is not guaranteed or endorsed by the publisher.

## Footnotes

1
http://gisaid.org

2
https://github.com/nf-core/viralrecon

3
https://www.gov.uk/government/publications/assessment-and-procurement-of-coronavirus-covid-19-tests/protocol-for-evaluation-of-rapid-diagnostic-assays-for-specific-sars-cov-2-antigens-lateral-flow-devices
